# Initial axon growth rate from embryonic sensory neurons is correlated with birth date

**DOI:** 10.1002/dneu.22743

**Published:** 2020-04-21

**Authors:** Anthony R. Horton, Alun M. Davies

**Affiliations:** ^1^ School of Biosciences Cardiff University Cardiff UK

**Keywords:** axon growth, birth date, chicken embryo, nodose sensory neuron, target distance

## Abstract

Axon growth rate from different populations of sensory neurons is correlated with the distance they have to grow to reach their targets in development: neurons with more distant targets extend axons at intrinsically faster rates. With growth of the embryo, later‐born neurons within each population have further to extend their axons to reach their targets than early‐born neurons. Here we examined whether the axon growth rate is related to birth date by studying the axon growth from neurons that differentiate in vitro from precursor cells isolated throughout the period of neurogenesis. We first showed that neurons that differentiated in vitro from different precursor cell populations exhibited differences in axon growth rate related to in vivo target distance. We then examined the axon growth rate from neurons that differentiate from the same precursor population at different stages throughout the period of neurogenesis. We studied the epibranchial placode precursors that give rise to nodose ganglion neurons in the chicken embryo. We observed a highly significant, threefold difference in axon growth rate from neurons that differentiate from precursor cells cultured early and late during the period of neurogenesis. Our findings suggest that intrinsic differences in axon growth rate are correlated with the neuronal birth date.

## 1 INTRODUCTION

In the developing vertebrate peripheral nervous system, newly differentiated neurons initially survive independently of neurotrophins when their axons are growing to their targets and become dependent on target‐derived neurotrophins for survival when their axons reach their targets (Davies, 1987). The timing of target encounter by growing axons depends on target distance and axon growth rate. If dissociated cultures of different populations of sensory neurons are established when their axons are starting to grow to their targets in vivo, initial axon growth rate is correlated with target distance; the further axons have to grow, the faster they grow. This was first and most clearly demonstrated for the placode‐derived cranial sensory neurons of the vestibular, geniculate, petrosal, and nodose ganglia in the chicken embryo (Davies, 1989). These neurons are born over the same period of development and start extending axons to their targets at the same time, but the distance they have to grow to reach these targets is markedly different. Neurons with more distant targets extend axons at faster rates both in vivo and in vitro than neurons with nearby targets. The finding that these differences in axon growth rate are observed from neurons grown as single cells suggested that axon growth is controlled by an intrinsic program in the early neurons that is independent of interaction with other cells (Davies, 1989). The locations of these neurons together with their central and peripheral projections at early stages of development are illustrated in this publication. A similar phenomenon has been described for the neural crest‐derived neurons of different dorsal root ganglia (DRG). While the differences in target distance are not as great for cranial ganglia, limb DRG neurons, which have the more distant targets in vivo, have significantly faster axon growth rates in vitro than thoracic DRG, which have closer targets in vivo (Lallemend et al., 2012).

Because neurons within any population are born over a period of development, with growth of the embryo the distance late‐born neurons have to extend axons to reach their targets is greater than that of early‐born neurons. To examine the possibility that initial axon growth rate is correlated with birth date, we focused on the neurons of the chicken embryo nodose ganglion. These sensory neurons extend central axons to targets in the hindbrain and peripheral axons to targets in the viscera, including distant targets in the gastrointestinal tract. Their axons have the greatest distance of any neuronal population to grow to reach their targets and take the longest time to get there. As such, they have the fastest initial axon growth rate of any population of developing neurons (Davies, 1989; Vogel & Davies, 1991). Because these neurons have the greatest rate of change of target distance throughout the period of neurogenesis, they are particularly suitable for studying whether initial axon growth rate is related to birth date. ^3^[H]‐thymidine labeling studies have shown that nodose neurons are born from precursor cells between Day 2 and Day 5 *in ovo* of the chick embryo (D'Amico‐Martel, 1982), Hamilton–Hamburger (HH) stages 13 to 26 (Hamburger & Hamilton, 1951).

While it would be possible to establish dissociated cultures of nodose ganglia throughout the period of neurogenesis, the results of such studies would be confounded because older ganglia contain a mixture of early‐born and late‐born neurons and an increasing proportion of neurons that had reached their targets and become dependent on BDNF for survival (Vogel & Davies, 1991). For this reason, we studied neurons that differentiated in cultures of the epibranchial placodal precursor cells at stages throughout the period of neurogenesis.

Epibranchial placodes are local thickenings of the surface ectoderm that give rise to the neurons of the geniculate, petrosal, and nodose ganglia. The location of these placodes has been accurately ascertained by histological studies, chick‐quail chimera studies, fate‐mapping studies, and by the sites of expression of specific epibranchial placode markers, especially chicken Sox3, whose epithelial expression becomes restricted to epibranchial placodes at an early stage in their appearance and is essential for the neurogenic capacity of these ectodermal regions (Abu‐Elmagd et al., 2001; D'Amico‐Martel & Noden, 1983; Ishii, Abu‐Elmagd, & Scotting, 2001; Tripathi, Ishii, Abu‐Elmagd, & Scotting, 2009). These studies have revealed that the respective neurogenic areas of the ectoderm for the geniculate, petrosal and nodose neurons have become clear and anatomically discrete by HH stage 17. By HH stage 19, the single nodose placode has become separated into two placodes in the third branchial arch adjacent to the third and fourth branchial clefts. The location of epibranchial placodes has been additionally confirmed by studies of epibranchial placodal neurogenesis and the location of addition markers (Kriebitz et al., 2009; Ladher, O'Neill, & Begbie, 2010; Watari‐Goshima & Chisaka, 2011). The development and precise location of neurogenic placodes has facilitated our study, which has revealed a clear correlation between the initial axon growth rate and neuron birth date.

## 2 RESULTS AND DISCUSSION

### 2.1 Neurons that differentiate in cultures of different epibranchial placode cells display differences in axon growth rate

To demonstrate that our experimental approach is viable, we first needed to show that neurons that differentiate in dissociated cultures of different placodes exhibit differences in axon growth rate. In cultures established from different early placode‐derived cranial sensory ganglia at the stage when axons are starting to grow to their targets the neurons extend axons at intrinsically different rates that are correlated with the distances that the axons have to grow to reach their targets in vivo (Davies, 1989). To ascertain whether neurons that differentiate from precursor cells in cultures of the corresponding epibranchial placodes also exhibit differences in axon growth rate, we studied axon growth rates from neurons that differentiate in dissociated cultures of the epibranchial placodes that give rise to the neurons of the geniculate, petrosal, and nodose ganglia, respectively.

**Figure 1:**
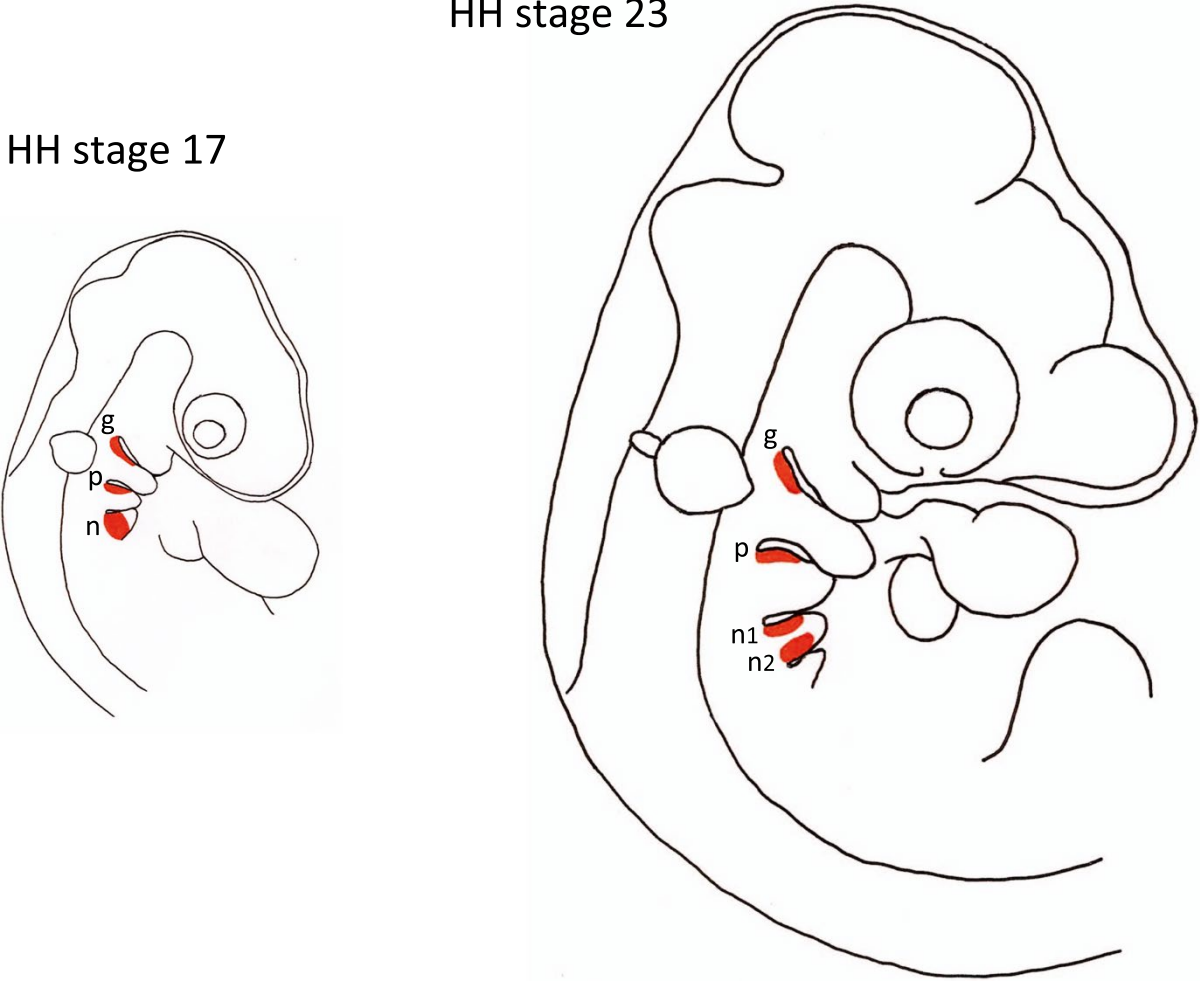
Schematic drawings of chicken embryo head showing the location of the epibranchial placodes that give rise to the neurons of the geniculate, petrosal, and nodose ganglia. The drawings illustrate the head at HH stages 17 and 23 and show in orange the epibranchial placodes that give rise to geniculate (g), petrosal (p), and nodose (n) neurons and the regions of ectoderm and immediately underlying mesoderm the were dissected in our study. The single nodose placode at HH stage 17 separates into two separate placodes (n1 and n2) by HH stage 19 and later stages [Color figure can be viewed at wileyonlinelibrary.com]

**Figure 2:**
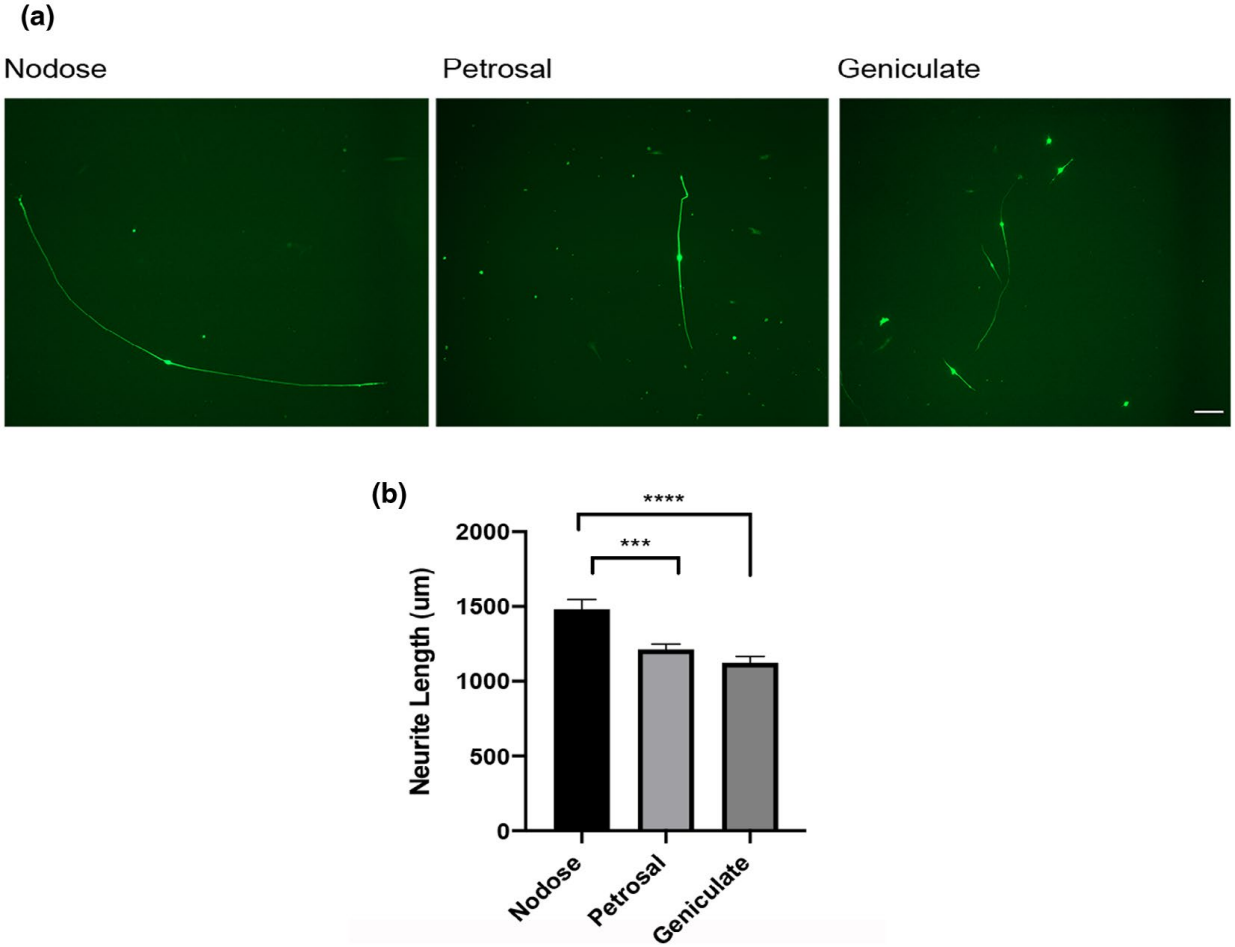
Neurons that differentiate cultured 1st, 2nd, and 3rd epibranchial placodes display differences in axon growth rate. (a) Representative images of β‐III tubulin‐positive neurons 24 hr after plating dissociated cultures of geniculate, petrosal, and nodose epibranchial placodes at HH stage 23. Scale bar = 100 µm. (b) Bar chart of axon lengths of neurons 24 hr after plating dissociated geniculate, petrosal and nodose epibranchial placode cultures at HH23. The combined results of five separate culture experiments are shown. Between 26 and 65 neurons (mean = 51) were measured of each kind in each experiment. Mean ± *SEM*, ***p < .001, unpaired *t*‐tests [Color figure can be viewed at wileyonlinelibrary.com]

The locations of the thickened regions of ectoderm that give rise to the geniculate, petrosal and nodose neurons are illustrated at HH stages 17 and 23 in Figure 1. Very fine tungsten needles were used to dissect these epibranchial placodes and the tissue immediately below the placodes, which contains migratory precursor cells from which geniculate, petrosal, and nodose ganglion neurons arise. Figure 2a shows images of representative neurons in low‐density dissociated cultures established from the geniculate, petrosal, and nodose placodes at HH stage 23. The axon lengths (distance between the growth cones of these bipolar neurons) of multiple neurons in these cultures after 24 hr in vitro is shown in Figure 2b. The length of axons of neurons that differentiated in dissociated cultures of the nodose placodes were longer than the axons of neurons that differentiated in dissociated cultures of the geniculate and petrosal placodes. These differences in axon length were highly significant. Although the axon lengths of neurons that differentiated in petrosal placode cultures were longer than neurons that differentiated in geniculate placode cultures, this difference did not reach statistical significance. Taken together, these findings suggest that the neurons that differentiate from different epibranchial progenitor cell populations in vitro exhibited similar differences in axon growth rate to neurons that have differentiated in vivo and have started extending axons to their targets (Davies, 1989). This suggests that the axon growth rate is already specified in epibranchial progenitor cells and that studies of axon growth rate from neurons that differentiate in progenitor cell cultures is a valid method for ascertaining whether axon growth rate and birth date are related.

The newly differentiated neurons in these cultures survived independently of BDNF and the addition of BDNF to the culture medium did not have any significant effect on axon growth rate (not shown). This is similar to the neurotrophin independence of sensory neurons in dissociated cultures of early placode‐derived cranial sensory ganglion neurons and the lack of BDNF on axon growth rate from these neurons previously reported (Davies, 1989).

### 2.2 Neurons that differentiate in cultures of early and late progenitors display different axon growth rates

**Figure 3:**
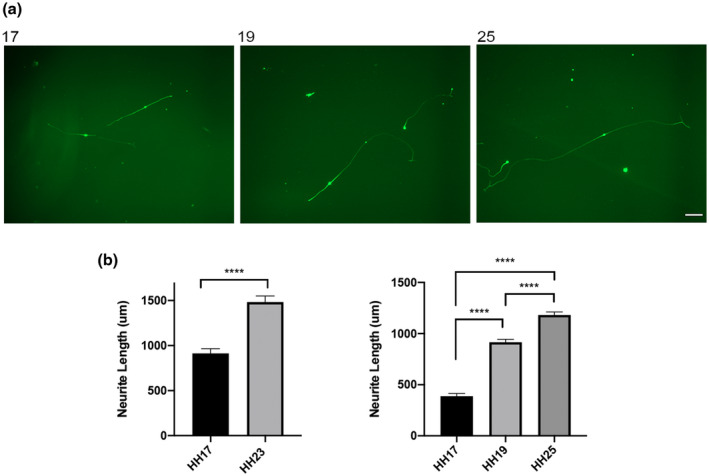
Neurons that differentiate in cultures of early and late third epibranchial placode display different axon growth rates (a) Representative images of β‐III tubulin‐positive neurons 24 hr after plating dissociated nodose epibranchial placode cultures at HH stages 17, 19, and 25. Scale bar = 100 µm. (b) Bar charts of axon lengths of neurons in 24 hr cultures of two sets of nodose epibranchial placode cultures at HH stages 17 and 23 (set one, *n* = five separate experiments, between 36 and 65 neurons measured at each stage per experiment) and HH stages 17, 19, and 25 (set two, *n* = four separate experiments, between 45 and 102 neurons measured at each stage per experiment). Mean ± *SEM*, ****p < .0001, unpaired *t*‐tests [Color figure can be viewed at wileyonlinelibrary.com]

Having demonstrated that neurons that differentiate in vitro from different progenitor cell populations exhibit differences in axon growth rate that are characteristic of neurons that arise from these progenitor populations in vivo, we were in a position to test whether neurons that differentiate from a particular progenitor cell population in vitro at stages throughout the period of neurogenesis exhibit differences in axon growth rate. Figure 3a shows images of representative neurons that have differentiated in low‐density dissociated cultures established from the nodose placodes at HH stages 17, 19, and 25. The axon lengths of multiple neurons in cultures after 24 hr in vitro is shown in the bar charts of Figure 3b. In two separate series of experimental studies there were marked and highly significant increases in axon length between HH17 and HH23 cultures (series one) and between HH17, HH19, and HH25 cultures (series two). These two separate series of results were obtained from extended periods of experimental work separated by more than 6 months. These robust and reproducible findings clearly demonstrate that initial axon growth rate is correlated with birth date. Sensory neurons that are generated early during the period of neurogenesis in a particular progenitor cell population when their axons have a relatively short distance to grow to reach their targets exhibit a slower axon growth rate than neurons generated later in development when the axons have further to grow to reach their targets. This implies the existence of a timing mechanism in the proliferating progenitor cell population that specifies initial axon growth rate.

In summary, we have discovered that neuron birth date is a novel parameter that affects axon growth rate from developing neurons. This is a previously unrecognized determinant of the correct timing of neuron/target encounter with the acquisition of trophic factor survival dependency, which is vitally important for integrating newly generated neurons in their changing environment. While our study has documented a novel developmental phenomenon in the early nervous system, the considerable experimental challenge of ascertaining the molecular basis of intrinsic differences in axon growth rate from neurons with generation time will have to await the development of suitable tools. The extremely small number of specific cells available for investigation precludes conventional biochemical approaches, which leaves targeting particular gene candidates as the most promising and precise way forward. However, while the early chicken embryo has experimental advantages that facilitated the discovery of intrinsic differences in initial axon growth rate related to target distance and birth date, the chicken presently lacks the necessary genetic tools to target particular genes. The first step will be to transfer the body of observations on early chicken sensory neurons to the mouse embryo.

Another pressing issue is the extent to which intrinsic differences in axon growth rate related to target distance occurs in other populations of developing neurons. The relative facility of studying initial axon growth rate from different populations of sensory neurons in vivo and in vitro has so far restricted study of initial intrinsic differences in axon growth rate to this class of neurons. It will be challenging to ascertain whether populations of other classes of neurons that project different distance to their targets, such as different populations of motoneurons or pyramidal neurons, also exhibit differences in initial axon growth.

Ascertaining the mechanisms that control of axon growth rate in development may have important implications for understanding axon regeneration following injury, especially the limited axon regeneration and consequent minimal functional recovery that occurs after CNS injury. Our discovery of a new parameter that affects the axon growth rate in development may have repercussions for research into axon regeneration following injury.

## 3 MATERIALS AND METHODS

### 3.1 Dissection

White Leghorn chicken eggs were incubated at 38°C in a forced‐draft incubator to the required stage of development. Embryos were staged according to the criteria of Hamburger and Hamilton (Hamburger & Hamilton, 1951). The epibranchial placodes and the immediately underlying mesenchyme, which contains neuron precursors that are migrating to the respective nascent geniculate, petrosal, and nodose ganglia, were dissected with very fine tungsten needles that had been sharpened electrolytically (Davies, 1995). Great care was taken not include any deeper tissue and any part of the nascent ganglia.

### 3.2 Cell culture

Dissected tissue was trypsinized, washed, and triturated as described previously (Buj‐Bello, Pinon, & Davies, 1994). The cells were plated in 35‐mm plastic tissue culture dishes (Nunclon) that had been pre‐coated with polyornithine (0.5 mg/ml, overnight) and laminin (20 μg/ml for 4 hr) at a very low density (between 200 and 500 cells per dish). The cells were grown in Ham's F14 medium plus 10% heat inactivated horse serum with or without recombinant BDNF.

### 3.3 Measurement of axon length

After 24 hr incubation in vitro, neurons were fixed for 10 min in 4% paraformaldehyde in 0.12 M phosphate‐buffered saline (PBS), washed three times in PBS, and blocked in 1% bovine serum albumin (Sigma), 0.1% Triton (Sigma) in PBS for 1 hr, then incubated with primary antibodies against neuron specific βIII‐tubulin (1:500, mouse‐monoclonal, MAb 1195, R&D Systems) at 4°C overnight. After washing, the neurons were incubated with polyclonal Alexa‐conjugated secondary antibodies (goat anti‐mouse Alexa‐488, Invitrogen) 1:500 for 1 hr at room temperature. Cells were then washed with PBS, and visualized using a Zeiss Axiovert 200 fluorescence microscope fitted with an AxioCam IcC5 camera and EC Plan Neofluoar 10× lens. Axon length was quantified using Fiji software with the plugin for Sholl Analysis (Schindelin et al., 2012), following neuronal reconstruction with the plugin Simple Neurite Tracer (Longair, Baker, & Armstrong, 2011).

## CONFLICT OF INTERESTS

The authors declare no competing or financial interests.

## AUTHOR CONTRIBUTIONS

A. Horton carried out the in vitro experiments and axon measurements and A. M. Davies did most of the dissections and wrote the manuscript.

## Data Availability

The data that support the findings of this study are available from the corresponding author upon reasonable request.
